# Comparative analysis of microRNA and messengerRNA expression profiles in plateau zokor testicular cells under reproductive suppression

**DOI:** 10.3389/fvets.2023.1184120

**Published:** 2023-05-19

**Authors:** Baohui Yao, Yukun Kang, Kang An, Yuchen Tan, Qiqi Hou, Degang Zhang, Junhu Su

**Affiliations:** ^1^Key Laboratory of Grassland Ecosystem (Ministry of Education), College of Grassland Science, Gansu Agricultural University, Lanzhou, China; ^2^Gansu Agricultural University-Massey University Research Centre for Grassland Biodiversity, Gansu Agricultural University, Lanzhou, China

**Keywords:** microRNA, spermatogenesis, testicular development, reproductive suppression, plateau zokor

## Abstract

**Introduction:**

Reproductive suppression is an adaptive strategy that affects the success rate and reproductive efficiency in animals, which in turn affects population continuation and evolution. However, no studies on the miRNAs in testicular development and spermatogenesis regulatory mechanisms under reproductive suppression have been reported.

**Methods:**

In this study, the differentially expressed (DE) miRNAs, miRNA–mRNA interaction network and function of the plateau zokor testicular cells of non-breeders and breeders during the breeding season were comprehensively analyzed by transcriptomics.

**Results:**

In total, 381 known and 94 novel miRNAs were determined. Compared with that in the breeders, 70 downregulated and 68 upregulated DE miRNAs were identified in the non-breeders. We predicted 1670 significant target mRNAs by analyzing the miRNA and mRNA expression profiles. According to the miRNA–mRNA interaction network, the target mRNAs of the DE miRNAs were related to testicular development and spermatogenesis. GO indicate that the target mRNAs were related to testicular development and spermatogenesis. KEGG indicate that pathways of target mRNAs enrichment related to testicular development, spermatogenesis, and energy metabolism. *PROK2* was determined as the target mRNA of rno-miR-143-3p.

**Discussion:**

Our study offers a basis for the regulatory mechanisms of miRNAs in testicular development and spermatogenesis in plateau zokor under reproductive suppression and offers a reference for reproductive regulation.

## Introduction

Animals have evolved to display diverse reproductive strategies in response to both abiotic and biotic factors, and reproductive suppression is an adaptive strategy that affects the success and efficiency of reproduction, which in turn influence survival and evolution (1, 2). Reproductive suppression refers to the inhibition or damage of normal reproductive development, reproductive physiology, or reproductive behavior in animals due to the impact of specific environmental factors or internal conditions, resulting in reduced or lost reproductive capacity (1, 2). Reproductive suppression manifests as delayed puberty or inhibition of the development of secondary sexual signs in adolescence (3, 4), delayed gonadal and gametic development (5), and reduced serum testosterone or estradiol levels (5, 6). Transcriptome analysis of the testicular cells of non-breeding naked mole rats (*Heterochephalus glaber*) under reproductive suppression revealed downregulation of the genes that regulate testicular development and spermatogenesis, such as those related to postmeiotic spermatogenesis (e.g., *PRM1*, *ODF3*, and *AKAP4*) (7), lipid biosynthesis processes and steroid hormone biosynthetic pathways (e.g., *CYP11A1*, *ABCG8*, and *SCARB1*) (8), and endocrine signaling (e.g., *SSTR3*, *TAC4*, and *CAMP*) (9).

In addition to mRNAs, non-coding RNAs play a major role in reproductive regulation. Sperm cells house different RNA families (including miRNA, piRNA, snoRNA, and snRNA), 7% of which are miRNAs (10). miRNA is non-coding RNA that is 19–24 nt long, was first found in Cryptomeria elegans to regulate gene expression by forming a complementary structure with the 3’UTR of the target mRNA. miRNAs play a critical role in several stages of spermatogenesis, including self-renewal and differentiation of spermatogonia stem cells (SSC), spermatocyte meiosis, spermatid maturation, and proliferation of Sertoli cells (11). Increased expression of let-7 downregulates *LIN28D* expression, inhibits *MYCN* and *CCDN1* expression, and promotes SSC differentiation (12). miR-224 regulates the self-renewal of mouse SSC by regulating *PLZF* and *GFRα1* (13). miR-34c upregulates the expression of meiotic regulatory genes (*STRA8*, *SCP3*, and *DAZL*) through the target gene *NANOS2* and promotes SSC differentiation and meiotic processes (14). The upregulation of miR-122 leads to the degradation of TNP2, which leads to sperm defects as TNP2 completes the compression and condensation of chromatin required for spermatogenesis (15). Additionally, miR-133b regulates the expression of *CCNB1* and *CCND1* through the target gene *GLI3* and regulates the multiplication of Sertoli cells (16). Therefore, the function of miRNAs in testicular development and spermatogenesis must be investigated to increase our understanding of the regulatory mechanisms underlying reproductive suppression.

Plateau zokors (*Eospalax baileyi*) are subterranean rodents residing in alpine grasslands and meadows (17). They are regarded as “ecosystem engineers,” in the grassland ecosystem of the Qinghai–Tibet plateau because at a normal population density they promote the soil nutrient cycle, improve soil structure, and increase plant species diversity. However, at high population density, their activities such as digging the soil to build nests and competing with livestock for forage aggravates grassland degradation and soil erosion (18). Therefore, the population density of the plateau zokors needs to be controlled to maintained environmental balance. Recently, we observed reproductive suppression in plateau zokors and that spermatogenic arrest occurred at the spermatogonia stage in non-breeder (19), and the expression of the genes involved in spermatogenesis was downregulated during the meiotic and postmeiotic stages in non-breeders compared with that in breeders (19). Hence, the corresponding mRNAs may inhibit testicular development and spermatogenesis in non-breeders, resulting in spermatogenic arrest (19). Although miRNAs regulate mRNA expression and play a major role in spermatogenesis in plateau zokors under reproductive suppression, their specific role in testicular development and spermatogenesis under reproductive suppression has not been well studied, which limits our understanding of the regulatory mechanisms underlying reproductive suppression. In our study, we analyzed the miRNA expression profiles in the testicular cells of non-breeding and breeding plateau zokors during the breeding season and integrated the data with the mRNA expression profiles of testicular cells obtained in our previous studies (19). We aimed to predict the miRNA–mRNA interaction network and identify central miRNA and target mRNA that regulate testicular development and spermatogenesis in plateau zokors under reproductive suppression. Our results would provide perspectives on the molecular regulatory mechanisms underlying reproductive suppression in animals.

## Materials and methods

### Animals

Plateau zokors were caught alive using tube traps at the end of April 2020. We collected the testes for total RNA extraction (19). We previously found that based on testis weight, serum hormones, hematoxylin and eosin staining, and immunohistochemistry data, the plateau zokors were divided into non-breeders (BSA) during the breeding season and breeders (BSB) during the breeding season (19). Firstly, by observation and palpation, males in the breeding period had relatively large testicles, and the bulge was observed in the abdominal groin. Secondly, the testicular weight and testicular coefficient of reproductive inhibited individuals were significantly lower than those of normal reproductive individuals. Finally, testosterone level was decreased and spermatogenesis was blocked in reproductive inhibited individuals plateau zokors (19).

### RNA sequencing

Three testicular samples each from the BSA and BSB groups were selected for transcriptome sequencing and sent to Beijing NovoGene (Beijing, China) (19).

### Analysis and prediction of miRNA

The small RNA reads were aligned with the reference genome assembly (*Nannospalax galili*) using Bowtie (20). Known miRNAs were identified using the miRBase database and mirdeep2 (21). Novel miRNAs were predicted using mirdeep2 and miREvo (22). We selected the differentially expressed (DE) miRNAs based on the conditions |log_2_foldchange| > 1 and p-adj value <0.05.

### Target mRNA prediction and enrichment analysis

To understand the underlying role of miRNAs in the testes, we predicted the target mRNAs of the DE miRNAs. The candidate target mRNA of the DE miRNA was predicted using miRanda (23) and RNAhybrid (24). To explore the underlying functions of the DE miRNAs, we performed Gene ontology (GO) functional analyses and Kyoto Encyclopedia of Genes and Genomes (KEGG) pathway enrichment analyses of the significant target mRNAs of the DE miRNAs. GO and KEGG enrichment analysis was performed for candidate target mRNAs using DAVID (25).

### miRNA–mRNA interactions network construction

We constructed an miRNA–mRNA interaction network by integrating the miRNA expression profile in the plateau zokor testicular cells with the mRNA expression profiles of testicular cells obtained in our previous studies (19). The miRNA–mRNA interaction network was integrated and visualized using Cytoscape 3.9.1 (26).

### Validation using real-time quantitative PCR (RT-qPCR)

Nine DE miRNAs (miR-34b-5p, miR-148b-3p, miR-450b-5p, miR-9a-5p, miR-15b-5, miR-499-5p, miR-429, miR-128-3p, and miR-143-3p) were randomly screened for RT-qPCR analysis. Detection of changes in the expression level of *PROK2*. The RT-qPCR primer sequence information are shown in Supplementary Table 1. Method reference: Zhang et al. (27).

### Dual-luciferase reporter assays

Fragments comprising PROK2-3′UTR (wild-type) of miR-143-3p and PROK2-3′UTR (mutant-type) of miR-143-3p and a positive control (miRNA inhibitor) were designed using Target Scan, and RNA22 and synthesized. The sequence information is listed in Table 1. Then, the *PROK2*-3′-UTR fragment containing the wild-type (WT-PROK2) and mutant (Mut-PROK2) sequences was cloned into the psiCHECK-2 vector. The miRNA (or miRNA inhibitor), recombinant vector, and Lipofectamine transfection reagent were mixed and transfected into HEK-293T cells. After 48 h of transfection, the activity of luciferase was determined.

**Table 1:** Sequence information of dual-luciferase reporter assays.

Gene name	Sequence information
Prok2 WT-rno-miR-143-3p	TGACTTACATTCTTGCAACAGAAGACAGCTCATTCATCTCACCCTGGGGAGGAGAGGTAT
Prok2 MUT-rno-miR-143-3p	TGACTTACATTCTTGCAACAGAAGTGTCCACAAAGTAGAGACCCTGGGGAGGAGAGGTAT

## Results

### Summary of sRNA libraries

To identify miRNAs in plateau zokor testicular cells, we constructed and sequenced six small RNA (sRNA) libraries designated as BSA1, BSA2, BSA3, BSB1, BSB2, and BSB3 libraries containing 11,923,149, 13,566,791, 11,189,808, 12,149,701, 15,583,297, and 15,463,645 raw reads, respectively. The low-quality sequences, erroneous reads, and reads with *N* > 10% were removed, and sequences shorter than 18 nt were discarded. In total, 11,270,471, 12,922,408, 10,649,515, 11,443,784, 14,781,619, and 14,679,946 clean reads were generated in the BSA1, BSA2, BSA3, BSB1, BSB2, and BSB3 libraries, respectively, and were used for further analysis (Table 2).

**Table 2:** Quality analyses of small RNA-seq data from testis of plateau zokor.

Sample	Total reads	Error rate	Q20	Q30	GC content	Low quality	Clean reads
BSA1	11,923,149	0.01%	99.12%	97.05%	48.64%	47,156 (0.40%)	11,270,471 (94.53%)
BSA2	13,566,791	0.01%	99.21%	97.15%	48.14%	49,467 (0.36%)	12,922,408 (95.25%)
BSA3	11,189,808	0.01%	99.16%	97.03%	48.70%	43,956 (0.39%)	10,649,515 (95.17%)
BSB1	12,149,701	0.01%	99.16%	96.94%	46.23%	47,721 (0.39%)	11,443,784 (94.19%)
BSB2	15,583,297	0.01%	99.13%	96.85%	46.84%	60,085 (0.39%)	14,781,619 (94.86%)
BSB3	15,463,645	0.01%	99.13%	96.86%	46.93%	59,489 (0.38%)	14,679,946 (94.93%)

Mapping the length-filtered sRNAs to the reference sequences (*N. galili*) covered 72.70–84.54% of the reads from six libraries (Table 3). Pearson’s correlation analysis showed that the correlation between each group of samples was strong, providing reliable data for the next step (Supplementary Table 2).

**Table 3:** The reads mapping to reference genome from sRNA-seq data in plateau zokor testes.

Sample	Total sRNA	Mapped sRNA
BSA1	10,425,930	8,617,297 (82.65%)
BSA2	12,009,131	9,835,766 (81.90%)
BSA3	9,340,806	7,896,996 (84.54%)
BSB1	11,179,811	8,127,892 (72.70%)
BSB2	14,469,154	10,880,553 (75.20%)
BSB3	14,466,684	10,845,803 (74.97%)

**Figure 1:**
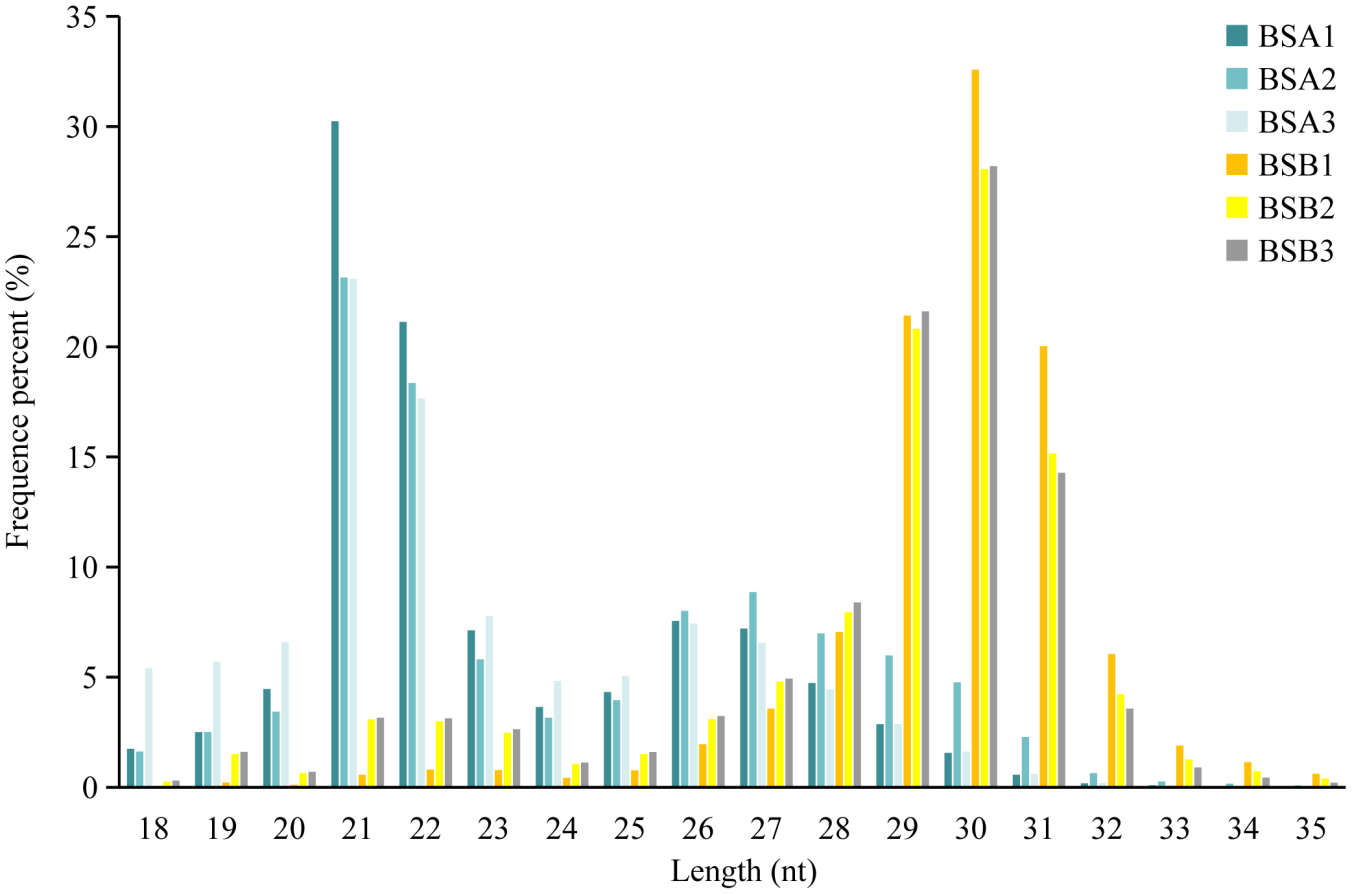
Size distribution and abundance of the sRNA libraries in plateau zokor testes.

Comparative analysis of the reading size distribution and abundance of six sRNA libraries revealed that the sRNA peaks for BSA1, BSA2, and BSA3 were 21–22 nt, whereas that for BSB1, BSB2, and BSB3 were 29–31 nt. sRNAs longer than 25 nt were Piwi-interacting RNAs (piRNAs), which have been reported to be abundant in the mature testicular cells in animals. The sRNA size distribution and abundance of BSA and BSB groups differed (Figure 1).

### Differentially expressed miRNAs in plateau zokor testicular cells

**Figure 2:**
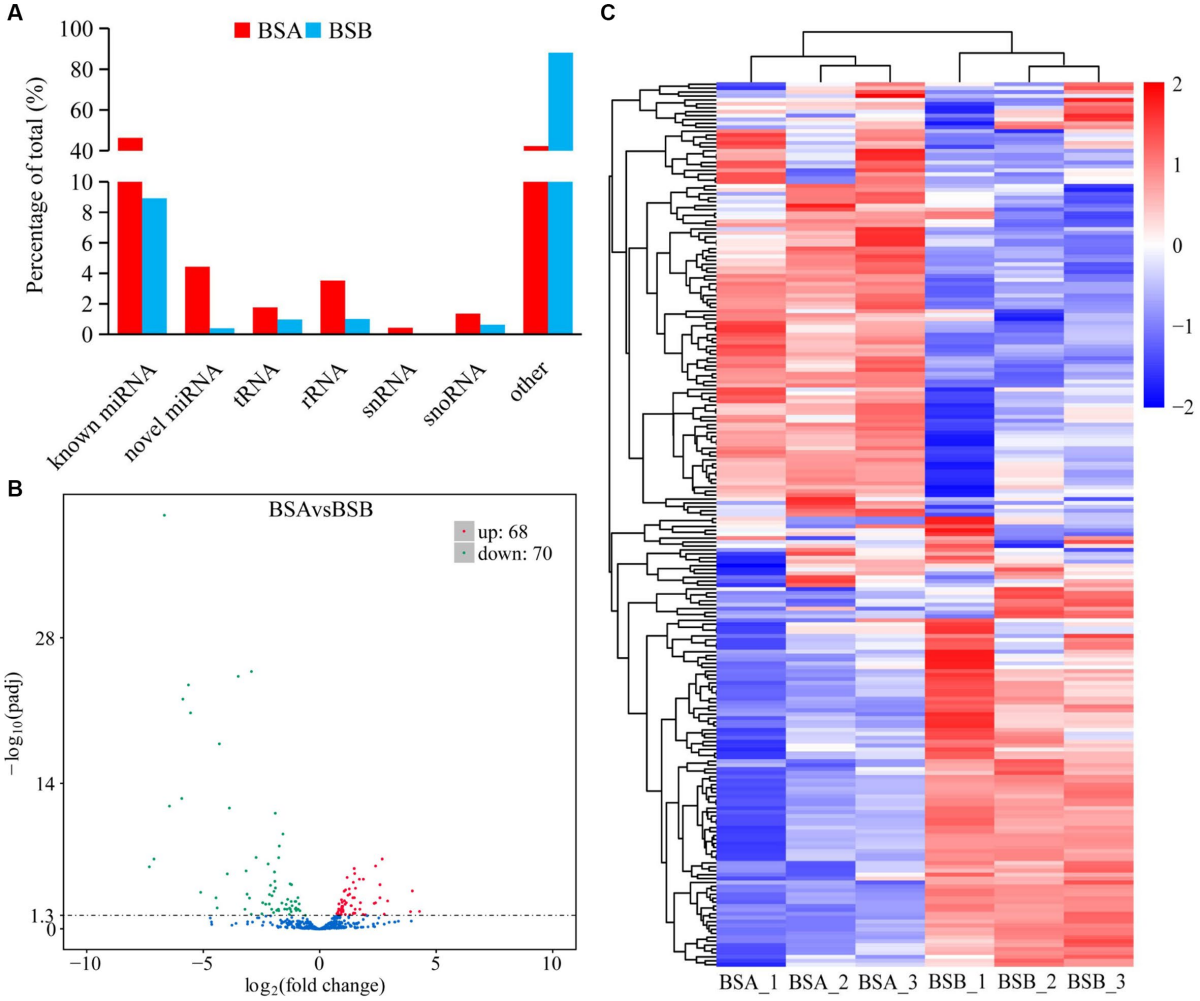
Differentially expressed miRNA in plateau zokor testes. **(A)** the percentage of small RNA types in plateau zokor testes; **(B)** The plot of the number of differentially expressed miRNA; **(C)** differentially expressed miRNA clustering graph.

The reads mapped to the reference sequence reads were aligned to the range of the sequences indicated in miRBase to obtain details of the known miRNAs. Novel miRNAs were predicted using miREvo and mirdeep2. Statistical analysis indicated that among all the sRNAs annotated in our study, the known miRNAs and other sRNA accounted for 46.29 and 42.20% of the total number of sRNA reads in BSA, respectively, whereas other sRNAs were the most abundant type in BSB, accounting for 88.04% of the small sRNA reads (Figure 2A). The other sRNAs in the BSB group may be piRNAs. Statistical results indicate that in total 475 miRNAs comprising 381 known miRNAs and 94 novel miRNAs from both BSA and BSB plateau zokor testicular cells were predicted in the six libraries (Supplementary Table 3).

We further analyzed the DE miRNAs derived from both the BSA and BSB testicular cells. The volcano plot shows 138 DE miRNAs (including 105 known and 33 novel miRNAs) from BSA and BSB testicular cells, of which 68 were upregulated and 70 were downregulated in the BSA compared with that in the BSB (Figure 2B; Supplementary Table 4). The top 10 DE miRNAs are listed in Table 4. The miRNA expression patterns in BSA and BSB were different from those in the cluster diagram (Figure 2C).

**Table 4:** Top 10 differentially expressed miRNA in plateau zokor testes.

sRNA	log2FoldChange	padj	sRNA	log2FoldChange	padj
Upregulated			Downregulated		
rno-miR-135a-5p	2.70	1.84E-07	rno-miR-15b-5p	−2.92	1.68E-25
rno-miR-200b-3p	2.60	5.41E-05	rno-miR-425-5p	−3.48	4.86E-25
rno-miR-193b-3p	2.41	8.72E-07	rno-miR-375-3p	−3.86	2.28E-12
novel_8	1.89	1.63E-05	rno-miR-130b-5p	−4.29	1.52E-18
novel_15	1.72	1.63E-05	rno-miR-449a-5p	−5.53	1.60E-21
rno-miR-181a-1-3p	1.52	4.59E-06	rno-miR-34b-3p	−5.62	3.27E-24
rno-miR-3,570	1.52	4.59E-06	rno-miR-34c-5p	−5.86	7.51E-23
novel_20	1.50	1.51E-06	novel_191	−5.91	2.74E-13
rno-miR-199a-3p	1.46	3.24E-05	novel_325	−6.44	1.49E-12
novel_26	1.19	1.09E-05	rno-miR-34b-5p	−6.65	1.54E-40

### Target mRNA prediction and enrichment analysis

In total, 4,860 miRNA–mRNA target pairs were predicted, and all 138 DE miRNAs were associated with the target mRNAs previously screened in the BSA and BSB groups (Supplementary Table 5). In total, 1,670 significant target mRNAs were identified (Supplementary Table 6).

**Figure 3:**
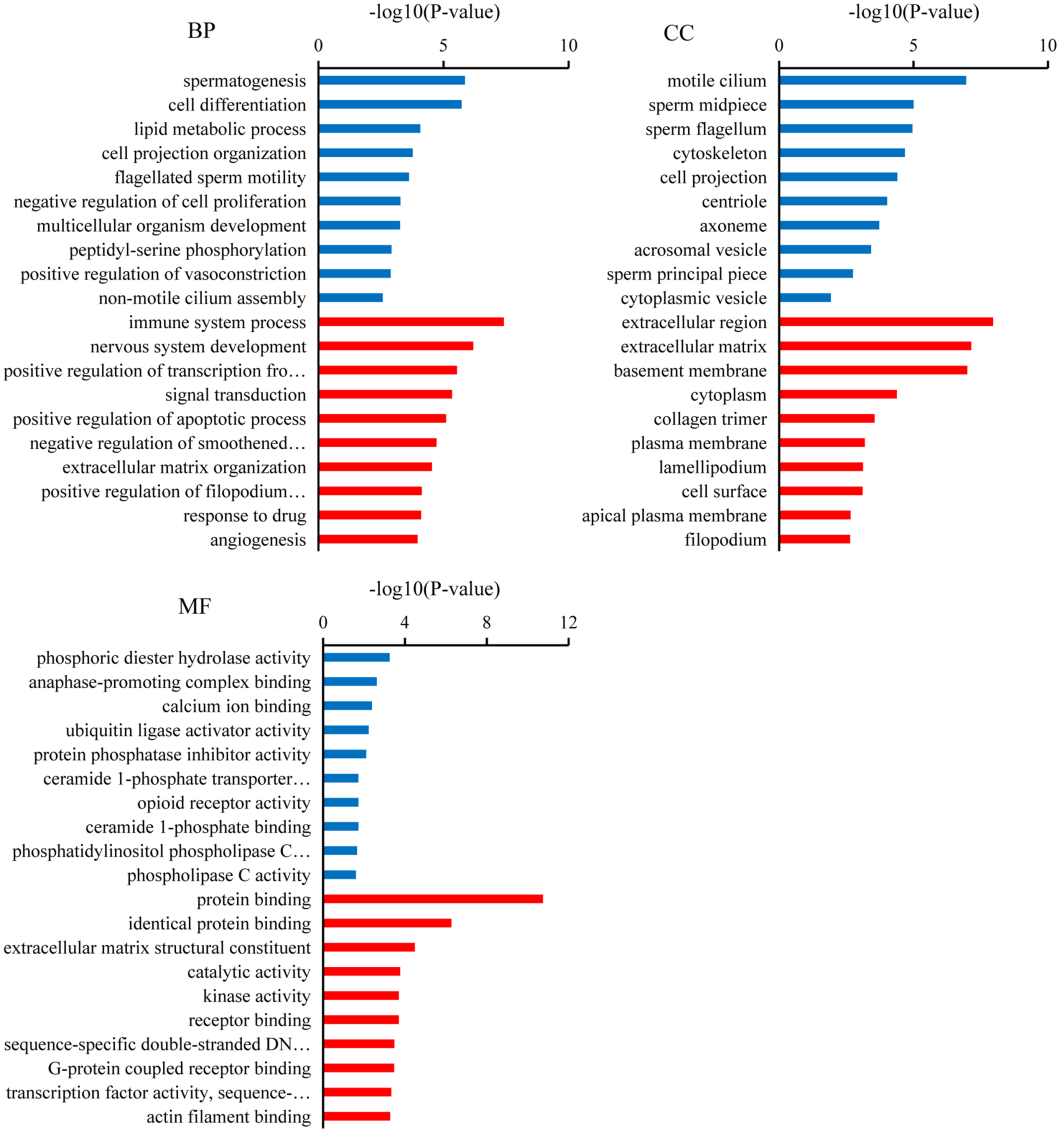
Gene ontology (GO) analysis of the candidate target mRNAs. A color code indicates overrepresentation (red) and underrepresentation (blue).

Gene ontology analysis indicated that the top ten biological processes in which the downregulated target mRNAs in BSA were significantly enriched compared with that in BSB included spermatogenesis, cell differentiation, cell projection organization, flagellated sperm motility, multicellular organism development, and lipid metabolic process (*p* < 0.05), and the upregulated target mRNAs were significantly enriched in positive regulation of apoptotic process, extracellular matrix organization, response to drug, and negative regulation of smoothened signaling pathway (*p* < 0.05). The top 10 cellular components in which the downregulated target mRNAs were significantly enriched include motile cilium, sperm midpiece, sperm flagellum, acrosomal vesicle, and sperm principal piece (*p* < 0.05), whereas the top 10 molecular functions in which the downregulated target mRNAs were significantly enriched include phosphoric diester hydrolase activity, anaphase-promoting complex binding, calcium ion binding, and ubiquitin ligase activator activity (*p* < 0.05; Figure 3).

**Figure 4:**
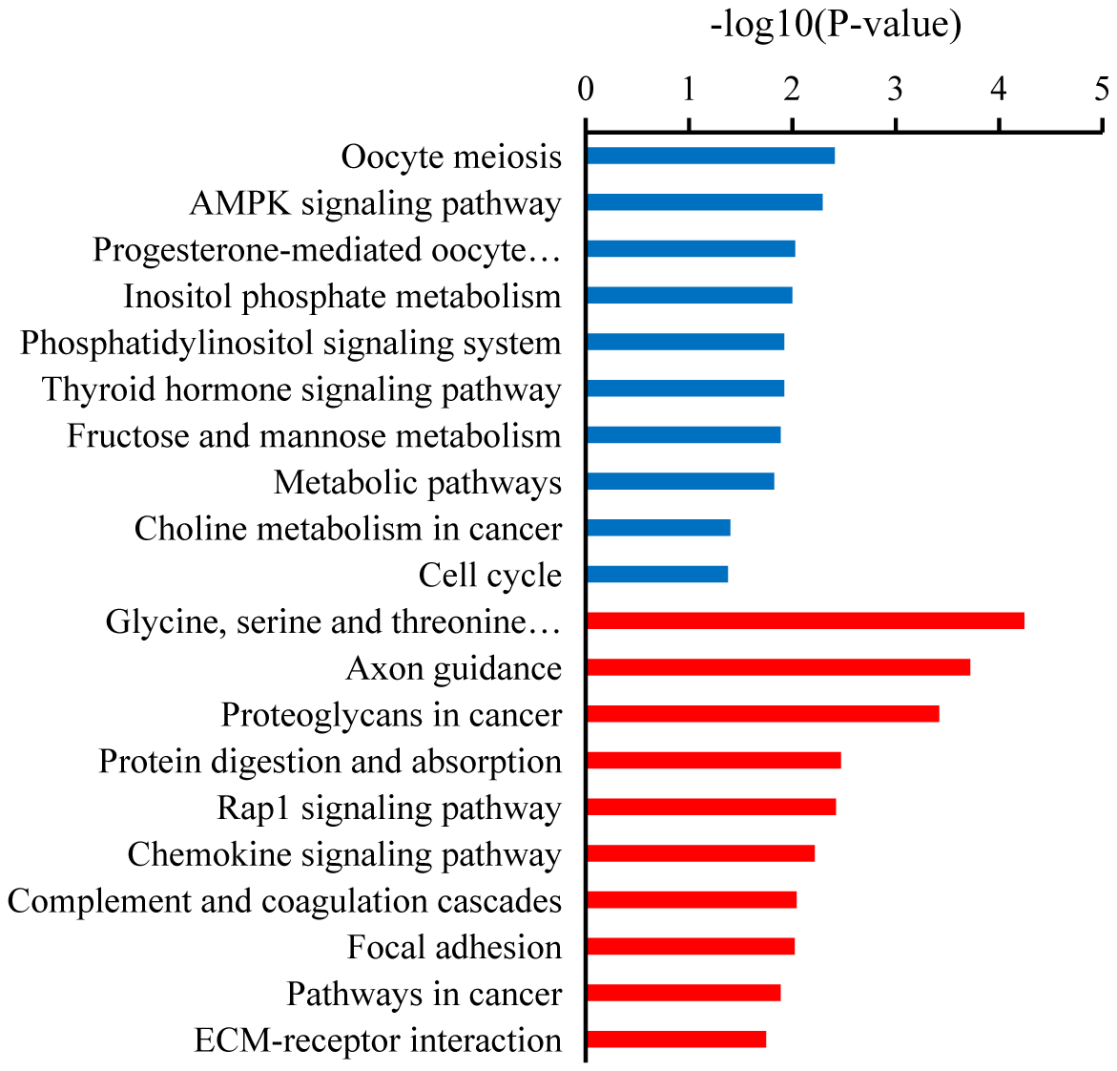
Kyoto Encyclopedia of Genes and Genomes (KEGG) enrichment pathways of the candidate target mRNAs. A color code indicates overrepresentation (red) and underrepresentation (blue).

Kyoto Encyclopedia of Genes and Genomes analysis showed that the downregulated target mRNAs in BSA were significantly enriched in oocyte meiosis, AMPK signaling pathway, cell cycle, progesterone-mediated oocyte maturation, metabolic pathways, thyroid hormone signaling pathway, inositol phosphate metabolism, fructose and mannose metabolism, and phosphatidylinositol signaling system (*p* < 0.05). The upregulated target mRNAs were significantly enriched in ECM-receptor interaction, focal adhesion, and chemokine signaling pathway, Rap1 signaling pathway (*p* < 0.05; Figure 4).

### miRNA–mRNA interactions network construction

**Figure 5:**
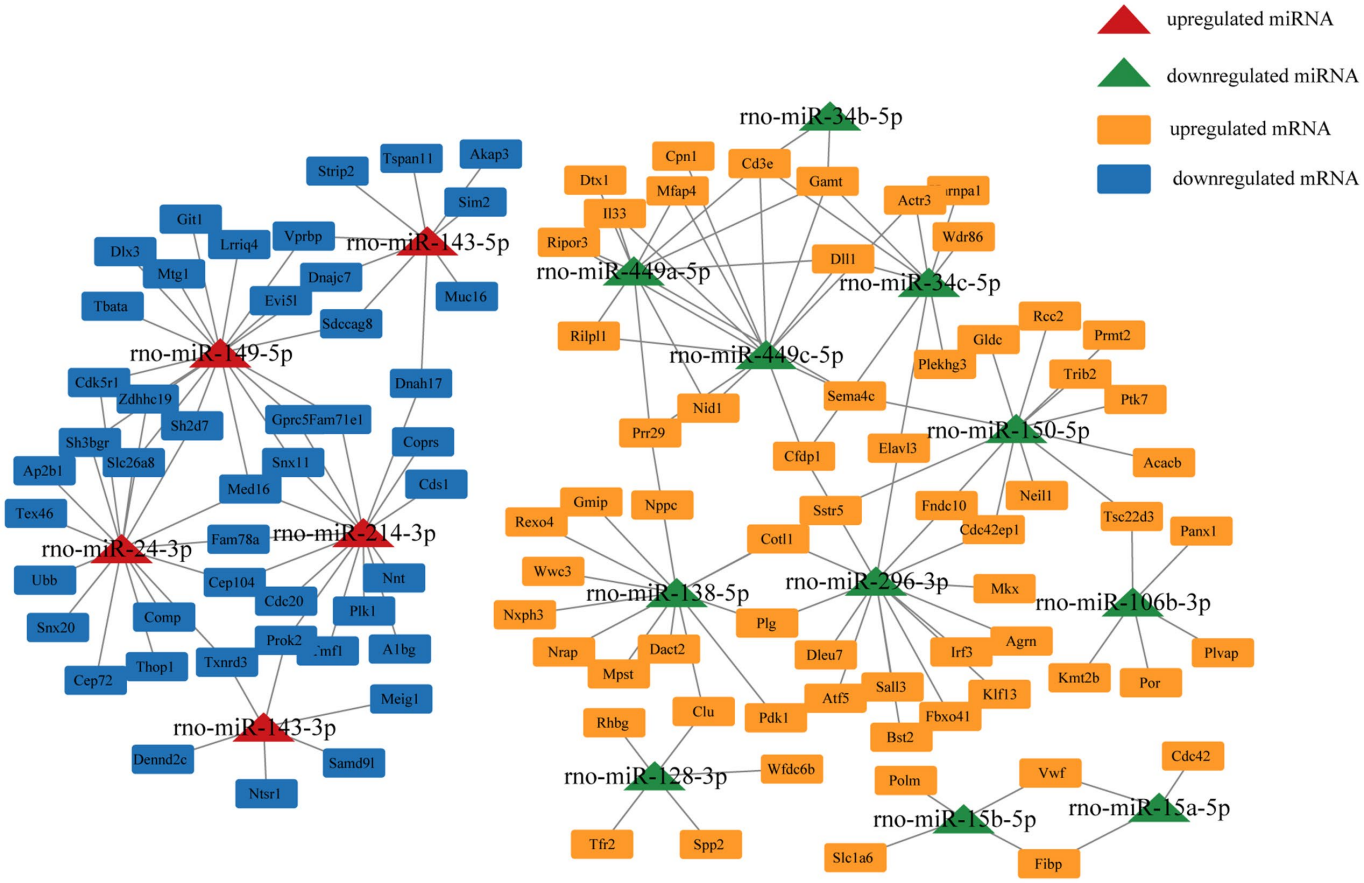
miRNA-Target mRNA regulatory networks in plateau zokor testes.

We selected miRNA–mRNA target pairs related to testicular development and spermatogenesis from 30,229 miRNA–mRNA target pairs and visualized them using Cytoscape (Figure 5). These key DE miRNAs (rno-miR-449a/c-5p, rno-miR-149-5p, rno-miR-296-3p, rno-miR-138-5p, rno-miR-143, and rno-miR-214-3p) significantly regulate mRNA expression in plateau zokor testicular cells. They also play a central role in spermatogenesis in plateau zokors.

### Validation using RT-qPCR

**Figure 6:**
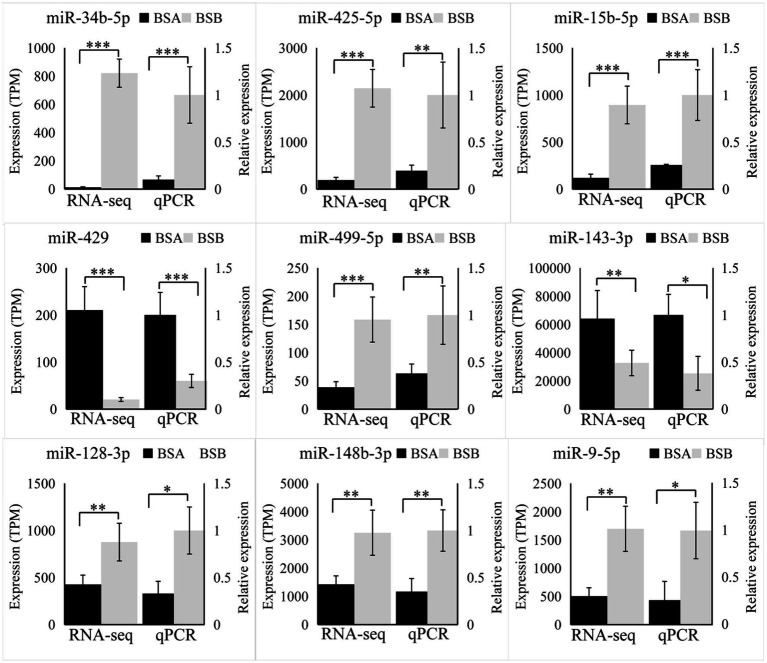
Validation by RT-qPCR. Data were shown as mean ± SD. The right axis represents gene expression levels verified by qRT-PCR, the left axis represents the expression levels in TPM units of RNA-seq. ^*^p < 0.05, ^**^p < 0.01, ^***^p < 0.001.

We randomly selected nine DE miRNAs (miR-34b-5p, miR-450b-5p, miR-9a-5p, miR-15b-5, miR-499-5p, miR-429, miR-148b-3p, miR-128-3p, and miR-143-3p) for RT-qPCR analysis. The variation trend of qRT-PCR results was consistent with that of RNA-seq data. Our results prove that RNA-seq data was accurate (Figure 6).

### rno-miR-143-3p targets PROK2

**Figure 7:**
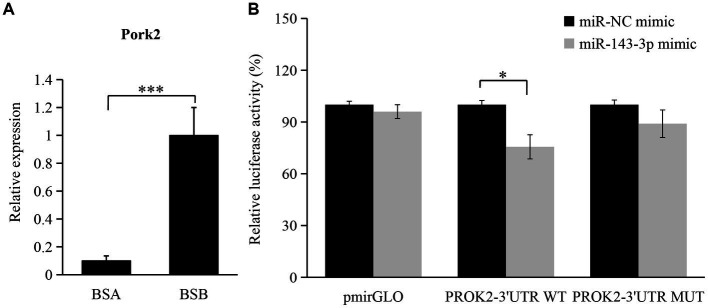
Validation of regulatory relationship between rno-miR-143-3p and PROK2. **(A)** Changes in the expression level of PROK2 in BSA and BSB; **(B)** miR-143-3p mimic was cotransfected with PROK2-3 ‘UTR WT or PROK2-3 ‘UTR MUT to detect the luciferase activity for 48 h. ^*^p < 0.05, ^***^p < 0.001.

According to GO and KEGG pathway analyses, the biological process and signaling pathway of target mRNA enrichment are associated with testicular development and spermatogenesis in plateau zokors. *PROK2* is closely related to spermatogenesis. The expression level of PROK2 in BSB is significantly higher than that in BSA (Figure 7A). According to a bioinformatics database, *PROK2* is a major target pair of miR-143-3p. Thus, we chose miR-143-3p and *PROK2* to validate the miRNA**–**mRNA regulatory relationship. We found that co-transfection of rno-miR-143-3p mimics for 48 h reduced the luciferase activity of *PROK2* receptors by 24.4% compared to the negative control (*p* < 0.05). The results show that rno-miR-143-3p directly targets PROK2-3′UTR (Figure 7B).

## Discussion

Studying the regulatory mechanisms of reproductive suppression is helpful in understanding the reproductive strategies in animals. In our study, we comparatively analyzed the miRNA expression profiles of plateau zokor testicular cells in both non-breeders and breeders and integrated them with the mRNA expression profiles obtained in previous studies. We determined 138 DE miRNAs, of which 68 were upregulated and 70 were downregulated in BSA and BSB testes. The top 10 DE miRNAs were mostly related to spermatogenesis and testicular development. GO and KEGG enrichment analyses suggested that the target mRNAs of the DE miRNAs were associated with spermatogenesis and testicular development. We predicted the miRNA–mRNA interaction network and identified the key miRNAs (rno-miR-449a/c-5p, rno-miR-149-5p, rno-miR-296-3p, rno-miR-138-5p, rno-miR-143, and rno-miR-214-3p) that may regulate testicular development and spermatogenesis in plateau zokors under reproductive suppression. We confirmed that PROK2 is a binding site for miR-143-3p.

Based on sRNA length distribution, we found that sRNAs from non-breeder testes were mainly distributed between 21 and 22 nt, which was consistent with the result that miRNA expression levels in the immature testes were higher than that in the mature testes (28). However, most sRNAs in the testes of the breeders were piRNAs with a length of 29–31 nt (29), and piRNAs were most abundantly expressed in male spermatogenesis. piRNAs are abundantly expressed in germ cells at meiosis I stage and are lost before the production of mature sperm (29, 30). Our RNA-seq data included piRNAs from the plateau zokor testicular cells and did not ignore the differences in miRNA and piRNA length distribution. This suggests that piRNA may be the key factor in suppressing spermatogenesis in plateau zokors under reproductive suppression.

In total, 138 (68 upregulated and 70 downregulated) DE miRNAs were determined in the six libraries. Among the top 10 DE miRNAs, miR-34b/c-5p, miR-34b-3p, miR-449a-5p, miR-130b-5p, miR-375-3p, miR-425-5p, and miR-15b-5p were significantly downregulated. Previous studies state that miR-34b/c is preferentially expressed in germ cells during the meiosis stage in mouse testes. Germ cells carry miRNA-34c as it is vital during the first meiosis stage of spermatogenesis (31). miR-449a is upregulated at the beginning of meiosis and is preferentially expressed in spermatocytes and haploid spermatids. miR-449 promotes proliferation and inhibits apoptosis of Sertoli cells by suppressing *PTEN* expression (32). Thus, reduced expression of miR-34/449 in the testes of non-breeders may reflect impaired spermatogenesis during meiosis. miR-130a activates *SMAD5* through the TGF-β-PI3K/AKT signaling pathway and promotes Sertoli cell growth in immature porcine (*Sus scrofa*) testes (33). The expression level of miR-375 in mature porcine testes was 5.4 times higher than that in immature porcine testes (28), leading to speculation that miR-375 affects porcine testicular development. miR-15a is significantly upregulated during the processes of spermatogonia to spermatocyte transformation (34), and its expression is significantly downregulated in dysspermia and asthenospermia (35). Thus, we conclude that the low expression of these miRNAs would affect testicular development and spermatogenesis in plateau zokors under reproductive suppression.

In our study, several GO terms associated with the male reproductive process were enriched, such as spermatogenesis, flagellated sperm motility, sperm midpiece, sperm flagellum, and sperm principal piece (36). Upregulated candidate target mRNA were significantly enriched in the apoptotic process. A dramatic increase in germ cell apoptosis occurs in some pathological conditions, which include idiopathic infertility in males. It has now been shown that the expression of apoptotic markers increases in the cryptorchidism testis which compromises fertility (37). Furthermore, the mRNAs that play a key role in male reproduction were significantly upregulated in the testicular cells of breeders, whereas these mRNAs were expressed at low levels in non-breeders. This reflects the limiting non-breeder testicular development and spermatogenesis. Similarly, KEGG analysis shows that most of the downregulated target mRNAs were enriched in spermatogenesis pathways, such as the oocyte meiosis, AMPK signaling pathway, progesterone-mediated oocyte maturation, thyroid hormone signaling pathway, fructose and mannose metabolism, and phosphatidylinositol signaling system. In non-breeders, a large number of pathways related to spermatogenesis were downregulated, which would limit testicular development and spermatogenesis, whereas in breeder, these pathways were upregulated, and testicular development and spermatogenesis were normal. Male meiotic stages I and II were significantly regulated by the oocyte meiotic pathway, which was crucial for spermatogenesis (38). Progestogen stimulates spermatogenesis through the progesterone-mediated oocyte maturation pathway, plays a key role in spermatogonial differentiation (39), and activates CatSper channels to accelerate sperm maturation in rhesus monkeys (*Macaca mulatta*) (40). In Sertoli cells, the AMPK signaling pathway regulates energy metabolism, junction complex stability, and cell proliferation (41) and is a key regulatory factor in the energy metabolism of germ cells, providing lactic acid and maintain spermatogenesis (42). Spermatogenesis was a process highly dependent on glycolytic metabolism for energy production, and endogenous synthetic fructose was the main source of energy for spermatozoa and may be important for fertility (43). Phosphatidylinositol signaling system play a central role in spermatogenesis, including the maintenance and proliferation and survival of germ cells and dynamic remodeling of cell adhesion in Sertoli cells (44). The thyroid promotes the eventual maturation of Sertoli cells by inhibiting their proliferation (45). Upregulated candidate target mRNA were significantly enriched in the chemokine signaling pathway and Rap1 signaling pathway. Studies have shown that some chemokines may have some effect on the germ cells involved in spermatogenesis. CXCL12 (C–C motif ligand 12) and its receptor type 4 (C–C receptor type 4) signal transduction regulate SSC activity, because failure of this axis leads to SSC loss and SSC *in vivo* (46). Interfering with Rap1 specifically in haploid cells results in an anomalous release of immature spermatids within the lumen of seminiferous tubuli and in low sperm counts; the loss of nondifferentiated cells correlated with impaired spermatid–Sertoli cell adhesion (47). Therefore, we hypothesized that these miRNAs participate in pathway through target mRNAs to regulate testicular development, spermatogenesis, and energy metabolism in plateau zokor testicular cells. The downregulation and upregulation of these signaling pathway in non-breeder testicular cells suggesting that testicular development, spermatogenesis, and energy metabolism in non-breeders are abnormal.

The core miRNA–target mRNA interaction network may reveal a regulatory relationship between testicular development and spermatogenesis in plateau zokors. We found that rno-miR-296-3p, rno-miR-138-5p, rno-miR-449a/c-5p, rno-miR-149-5p, rno-miR-143, and rno-miR-214-3p may be key miRNAs that regulate testicular functional development in the plateau zokors. Previous research have indicated that miR-296 as a tumor suppressor and cell motion suppressor, was upregulated in mature rhesus monkey testes (48). miR-138-5p targeted *CASPASE3* through the BCL2 signaling pathway and inhibited testicular cell apoptosis (49). miR-138-5p, miR-449, and miR-296-3p were down-regulated in the testes of non-breeders, which may damage spermatogenesis. The upregulation of miR-214 suppressed the target *TCP11*, which is a testes-specific gene that regulates haploid spermatids in inducing proper vitality in mature sperm (50). miR-149-5p regulates the multiplication and apoptosis of ovarian cancer cell (51). Nevertheless, the potential mechanism of action of rno-miR-149-5p in the testis remains unclear. miR-143-3p plays a tumor suppressive role in multiple of malignancies (e.g., liver cancer, bladder cancer, and ovarian cancer) (52). miR-143-3p suppressed cancer cell proliferation and migration in the ovary by regulating *TAK1* in ovarian cancer cells (53). miR-143-3p was expressed in porcine testes at various developmental stages, suggesting it was a key regulator of spermatogenesis (54). Compared with that in patients with obstructive azoospermia, miR-143-3p was upexpressed in human spermatogonia of patients with non-obstructive azoospermia, which led to spermatogenesis failure. *SMAD3* was the predicted target gene of miRNA-143-3p (55). *SMAD3* belongs to the *SMAD* superfamily and is transformed during TGF-β activation, which is crucial for the proliferation and differentiation of spermatogonia (56). These findings support our inference that upregulated miR-149-5p, miR-143, and miR-214-3p in the testes of non-breeders may impair spermatogenesis.

Additionally, PROK2 was a secreted protein involved in cell migration, proliferation, and apoptosis. Under normal physiological conditions, *PROK2* was upexpressed in the testes and was located in primary spermatocytes. *PROK2* knockout mice showed impaired male sexual development and infertility, as evidenced by the absence of spermatocytes and spermatids in the seminiferous tubule (57, 58). Therefore, *PROK2* is essential for regulating testicular development and spermatogenesis in animals. Bioinformatics and dual luciferase reporter analyses show that the 3′UTR of *PROK2* matched the seed sequence of miR-143-3p, which downregulates *PROK2* expression. However, the hypothesis that miR-143-3p regulates testicular development and spermatogenesis in plateau zokors by targeting *PROK2* remains to be elucidated.

## Conclusion

Our study provides the first comprehensively analyze miRNAs and mRNAs expression profiles during testicular development and spermatogenesis in plateau zokors. We identified central miRNA and target mRNA associated with testicular development and spermatogenesis in plateau zokors under reproductive suppression. Our results provide new perspectives on testicular development and spermatogenesis and help understand and illustrate the molecular regulatory mechanisms underlying reproductive suppression in plateau zokors. In particular, we confirmed the binding site of miR-143-3p on *PROK2*. In future, we plan to study the function of miRNAs in testicular development under reproductive suppression and elucidate the mechanism underlying spermatogenesis in plateau zokors to provide additional information for understanding the regulatory mechanisms of reproductive suppression in testicular development and spermatogenesis. We hope our findings will advance the overall understanding of reproductive suppression in mammals.

## Data availability statement

The datasets presented in this study can be found in online repositories. The names of the repository/repositories and accession number(s) can be found at: https://ngdc.cncb.ac.cn/gsa/browse/CRA008119, PRJCA010603.

## Ethics statement

The animal study was reviewed and approved by Animal Ethics Committee of Gansu Agricultural University (approval number GAU-LC-2020-014).

## Author contributions

BY and JS did the data analysis and wrote the manuscript. BY, YK, KA, YT, QH, and DZ performed investigation and collected the samples. BY, YK, KA, YT, and QH performed the formal analysis, methodology, and software. JS did the project administration and revised the manuscript. All authors contributed to the article and approved the submitted version.

## Funding

This research was funded by the National Natural Science Foundation of China (32272566), the “Fuxi Talent” Plan (Gaufx-02J03) of Gansu Agricultural University, and the Industrial Support Program Project (2022CYZC-47) of Gansu Provincial Education Department.

## Conflict of interest

The authors declare that the research was conducted in the absence of any commercial or financial relationships that could be construed as a potential conflict of interest.

## Publisher’s note

All claims expressed in this article are solely those of the authors and do not necessarily represent those of their affiliated organizations, or those of the publisher, the editors and the reviewers. Any product that may be evaluated in this article, or claim that may be made by its manufacturer, is not guaranteed or endorsed by the publisher.
